# Restoration of *klotho* gene expression induces apoptosis and autophagy in gastric cancer cells: tumor suppressive role of klotho in gastric cancer

**DOI:** 10.1186/1475-2867-13-18

**Published:** 2013-02-21

**Authors:** Biao Xie, Jianping Zhou, Guoshun Shu, Dong-cai Liu, Jiapeng Zhou, Jinhui Chen, Lianwen Yuan

**Affiliations:** 1 Departemt of Geriatric Surgery, Second Xiangya Hospital, Central South University, Changsha, Hunan 410011, China; 2 Department of General Surgery, 8th Changsha Hospital, Changsha, Hunan, 410015, China

**Keywords:** Klotho, Gastric cancer, Apoptosis, Autophagy

## Abstract

**Background:**

The loss of tumor suppressor gene expression is involved in the carcinogenesis of gastric cancer (GC). *Klotho* is a recently identified tumor suppressor gene that epigenetically inactivated in gastric cancer. However, the signaling pathways involved in the suppressive role of klotho have rarely been reported in gastric cancer. In this study, we investigated the involvement of klotho in gastric cancer cell proliferation, apoptosis, and autophagy as well as the associated signaling.

**Methods:**

Methylation of *klotho* gene promoter in GC-7901, MNK-45 and AGS gastric cancer cells as well as GES-1 normal gastric epithelial cells was detected by bisulfate-based PCR. Restoration of *klotho* gene expression was established by applying a demethylating agent and delivering *aklotho* gene expression vector into GC-7901 cells. Cell viability was measured by CCK-8 assay. Cell apoptosis and cycling were analyzed by flow cytometry. Autophagy was measured by detecting LC3-I and LC3-II expression. Protein levels and phosphorylation were measured by Western blot assay.

**Results:**

Methylation of *klotho* gene promoter and expression of the *klotho* gene were detected in GC cells. Restoration of *klotho* gene expression significantly inhibited cell proliferation, induced cell apoptosis, and increased LC3-I/LC3-II expression in GC cells. Restoration of *klotho* gene expression downregulated the phosphorylation levels of IGF-1 receptor, IRS-1, PI3K, Akt, and mTOR proteins. Both apoptosis and autophagy inhibitors blocked klotho-induced apoptosis and autophagy.

**Conclusion:**

Klotho is a tumor suppressor in gastric cancer, which regulates IGF-1R phosphorylation and the subsequent activation of IRS-1/PI3K/Akt/mTOR signaling, tumor cell proliferation, apoptosis, and autophagy.

## Introduction

Gastric cancer (GC) is the fourth most prevalent cancer and the second leading cause of cancer-related deaths worldwide [1]. GC is a complex disease involving numerous genetic and epigenetic alterations. Although *TP53* is one of the earliest reported frequently mutated tumor suppressor genes in primary GC, a growing number of genetic and epigenetic alterations in other tumor suppressors have been reported to be involved in the carcinogenesis of GC [2]. For example, mutation and promoter methylation of *p16* and phosphatase and tensin homolog (PTEN) tumor suppressor genes have also been investigated in gastric cancer. Few mutations in these two genes have been found. However, the promoter regions of *p16*, but not *PTEN*, exhibit frequent methylation [3]. Recently, the *klotho* gene has been demonstrated to be a novel tumor suppressor gene that is epigenetically inactivated in GC. Ectopic expression of *klotho* gene inhibited the growth of GC cells [4]. However, the signaling involved in the tumor suppressive role of klotho protein in GC has not been elucidated.

Klotho has been demonstrated to function as a tumor suppressor in several tumors. For example, klotho is observed to induce cell apoptosis and inhibit tumor growth through inhibiting insulin/ insulin-like growth factor-1 (IGF-1) signaling [5,6]. Tyrosine phosphorylation of the insulin/IGF-1 receptors induces cytoplasmic binding of insulin receptor substrate 1 (IRS-1) to these receptors and phosphorylation of multiple tyrosine residues of IRS-1 itself. This enables IRS-1 to activate several signaling pathways, including the PI3K (phos-phoinositide 3-kinase) / Akt / mTOR signaling and MAP kinase pathways. A number of studies revealed that insulin/IGF-1 and PI3K/Akt/mTOR signaling pathways are involved in the carcinogenesis of GC through inhibiting cell apoptosis [4,7]. We therefore proposed that klotho may inhibit IGF-1 signaling, and subsequently induce apoptosis in GC cells through downregulating PI3K-Akt-mTOR signaling in GC.

Autophagy is a mode of type II programmed cell death and is thought to be the crucial way to kill apoptosis-resistant tumor cells [8]. Autophagy begins with the formation of an autophagosome, which fuses with the lysosomal membrane to deliver its contents, such as toxins and damaged cellular components, for degradation [9]. During autophagosome formation, the microtubule-associated protein light chain 3 I (LC3-I) is conjugated to phosphatidylamine to form LC3-phosphatidylamine, termed LC3-II. LC3-II then translocates to the autophagosome membrane, the process of which is essential for autophagosome formation [9,10]. Therefore, a decrease in LC3-I and increase in LC3-II levels are markers reflecting the activation of autophagy. A number of studies have reported that autophagy signaling can be activated by multiple signaling pathways [8]. There is increasing evidence that tumor suppressor genes promote autophagy while oncogenes inhibit autophagy [11]. We therefore hypothesized that the *klotho* gene might also regulate autophagy in GC.

In this study, we investigated the involvement of klotho in GC cell apoptosis and autophagy as well as the associated signaling by delivering *klotho* gene expression vector into two GC cell lines. Our study provided the evidence for klotho’s regulation of signaling involved in cell survival, proliferation, and apoptosis in GC.

## Results

### Difference in klotho gene expression and promoter methylation between gastric cancer and normal cells

**Figure 1:**
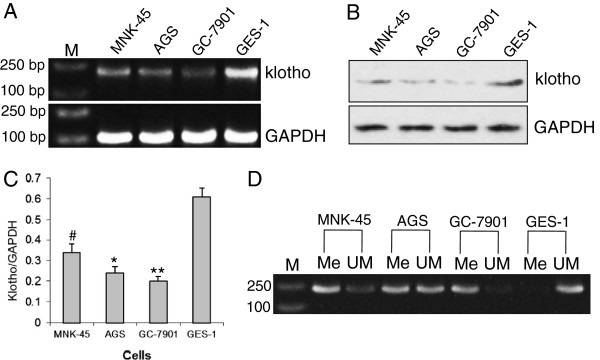
***Klotho *gene expression and methylation. A**) RT-PCR detection of *klotho *gene mRNA expression in GES-1 normal gastric epithelial cells and, MNK-45, AGS, and GC-7901 gastric cancer cells. **B**) Western blot of klotho protein expression. **C**) The relative klotho protein levels in **B**). **D**) Methylation of *klotho* gene promoter. The methylated (Me) and unmethylated (UM) *klotho *gene promoter fragments were amplified from bisulfite-treated genomic DNA by PCR. M: DNA ladder.

The mRNA expression of *klotho* gene was detected by RT-PCR and obviously lower klotho expression was observed in MNK-45, AGS, and GC-7901 gastric cancer cells than in the GES-1 normal gastric epithelial cells (Figure 1A). Western blot also showed lower klotho protein level in tumor cells than in normal cells (Figure 1B, C). The bisulfate-based PCR method was applied to examine the CpG methylation of *klotho* gene promoter. As shown in Figure 1D, the tested CpG site exhibited almost full methylation in GC-7901 cells, partial methylation in MNK-45 and AGS cells, but almost no methylation in GES-1 normal gastric epithelial cells.

### Restoration of *klotho* expression by a demethylating agent increases tumor cell apoptosis and authophagy

**Figure 2:**
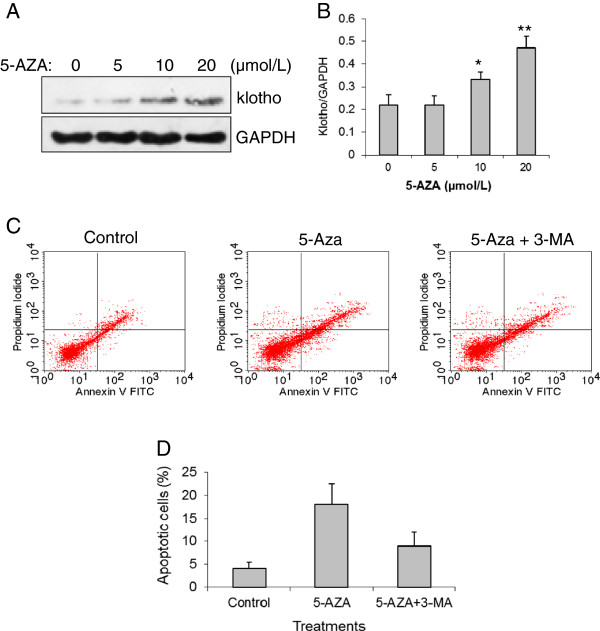
**The role of demethylating agent and autophagy inhibitor on klotho protein expression and apoptosis. A**) Representative Western blot of klotho protein expression. Demethylating agent 5-Aza upregulates klotho levels in a dose-dependent manner. **B**) Relative klotho protein levels in **A**). ^*^p < 0.01, ^**^p < 0.001 *vs.* 0 μmol/L of 5-Aza group. N = 5. **C**) Flow cytometry of apoptotic cells in GC-7901 cell under treatment of demethylating agent 5-Aza and autophagy inhibitor 3-MA. **D**) The percentage of apoptotic cells under treatment of 5-Aza and 3-MA. ^*^p < 0.01 *vs. *control; ^#^p < 0.05 *vs. *5-Aza treatment. N = 5.

**Figure 3:**
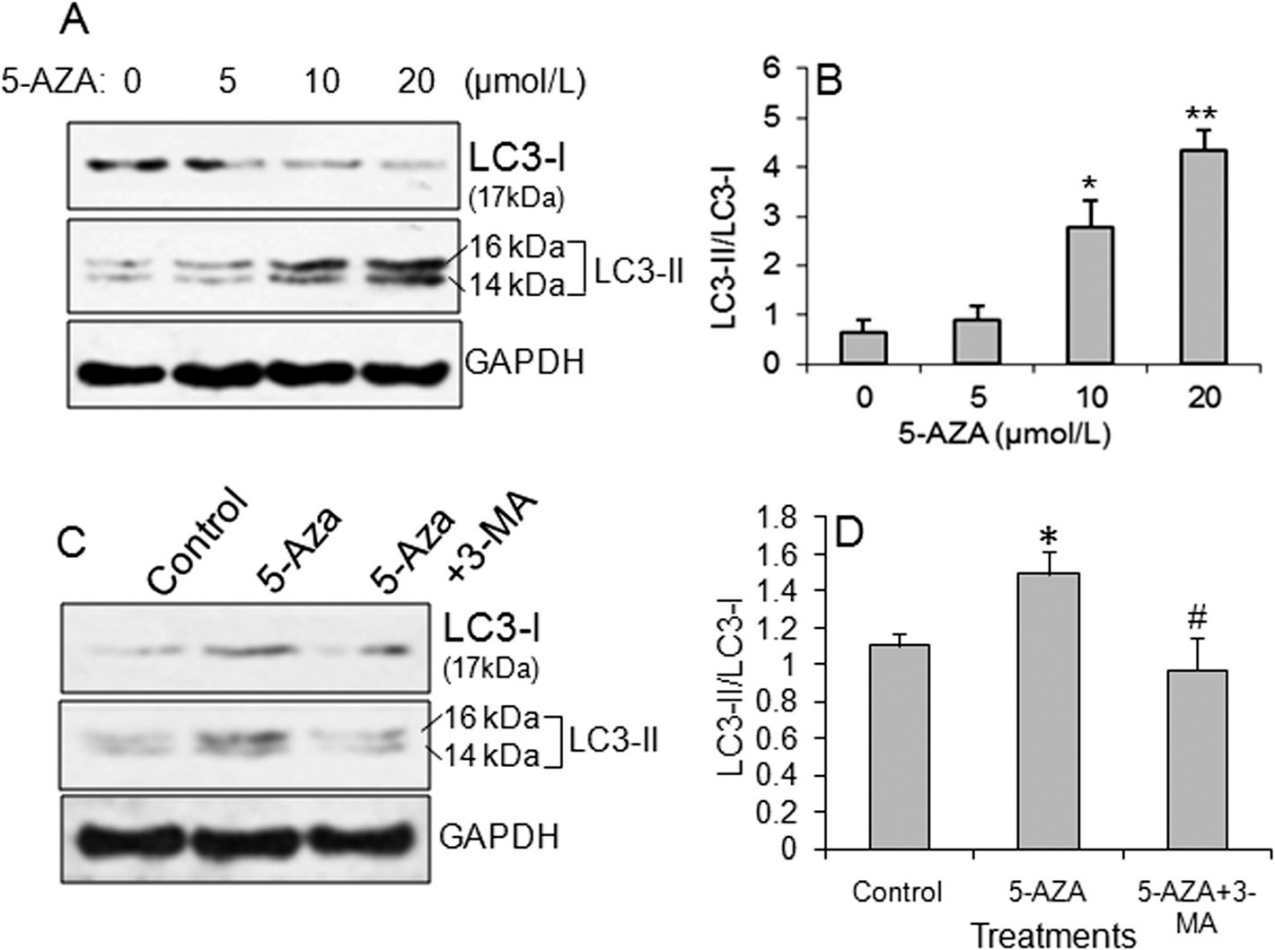
**The role of demethylating agent and autophagy inhibitor on autophagy. A**) Representative of Western blot of LC3-I and LC3-II protein expression. Demethylating agent 5-Aza upregulated LC3-II and downregulated LC3-I levels in a dose-dependent manner. **B**) Relative LC3-II/LC3-I ratio in **A**). ^*^p < 0.01, ^**^p < 0.001 *vs. *0 μmol/L of 5-Aza group. N = 5. **C**) Representative of Western blot of LC3-I and LC3-II protein expression under treatment of 5-Aza with or without 3-MA treatment. **D**) Relative LC3-II/LC3-I ratio in **C**). ^*^p < 0.01, ^**^p < 0.001 *vs. *0 μmol/L of 5-Aza group. N = 5.

Among the 3 tested gastric cancer cells, GC-7901 showed the lowest expression of *klotho* mRNA and protein and highest level in methylation of *klotho* promoter. Therefore, GC-7901 cells were chosen for further tests. As shown in Figure 2, DNA demethylating agent 2^′^-deoxy-5-azacytidine (5-Aza) increased klotho protein level in a dose-dependent manner (Figure 2A, B). The flow cytometry assay revealed that 5-Aza can restore *klotho* gene expression and significantly induce cell apoptosis in GC-7901 cells (Figure 2C, D). We further tested whether a DNA demethylating agent could affect autophagy and whether autophagy inhibitor (3-MA) could block it. GC-7901 cells were treated with 5, 10, and 20 μmol/L of 5-Aza for 12 hrs. 5-Aza dose-dependently decreased LC3-I level and increased LC3-II level (Figure 3A) with a significant increase in LC3-II/LC3-I ratio (Figure 3B), suggesting that a demethylating agent could significantly increase autophagy in gastric cancer cells. To test whether 3-MA could block the effect of 5-Aza in inducing autophagy, GC-7901 cells were incubated with 10 μmol/L of 5-Aza and 10 mM of 3-MA for 8 hrs. Results showed that 10 mM of 3-MA significantly blocked the role of 10 μmol/L of 5-Aza in inducing LC3-II expression and the ratio of LC3-II/LC3-I (Figure 3C, D).

### The signaling changes in GC-7901 cells subjected to demethylating agent and autophagy inhibitor treatment

**Figure 4:**
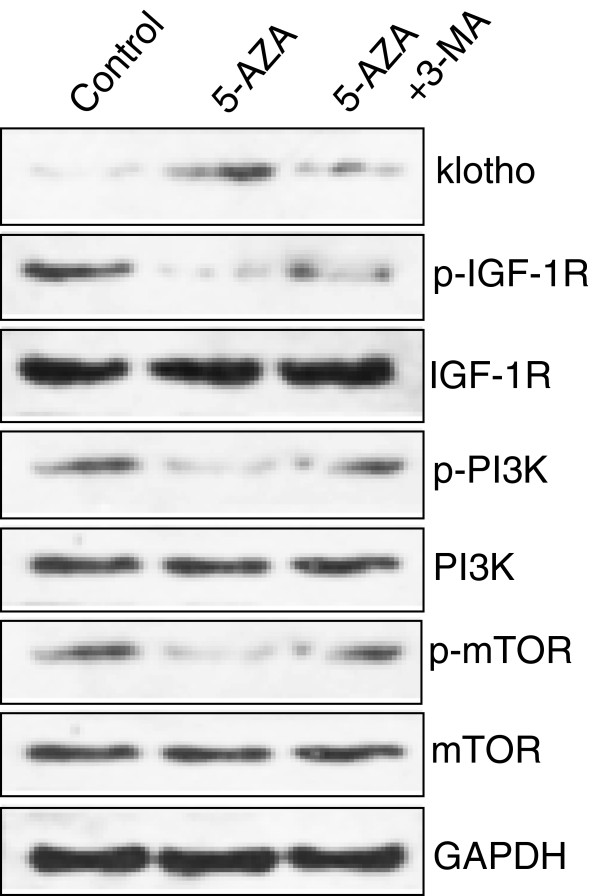
**The role of demethylating agen and autophagy inhibitor on protein expression and phosphorylation. **In GC-7901 cells, 5-Aza increased the level of klotho and decreased the levels of phospho-IGF-1R, phospho-PI3K, and phospho-mTOR, but had no effect on IGF-1R, PI3K, and mTOR levels. 3-MA significantly normalized the effect of 5-Aza.

The total protein and phosphorylated protein in demethylating agent and autophagy inhibitor-treated GC-7901 cells were detected by specific antibodies. Western blot showed that 5-Aza significantly increased the level of klotho and decreased the levels of phospho-IGF-1R, phospho-PI3K, and phospho-mTOR proteins, but had no effect on IGF-1R, PI3K, and mTOR protein levels (Figure 4). 3-MA significantly blocked the effect of 5-Aza (Figure 4).

### The signaling involved in the inhibitory role of klotho

**Figure 5:**
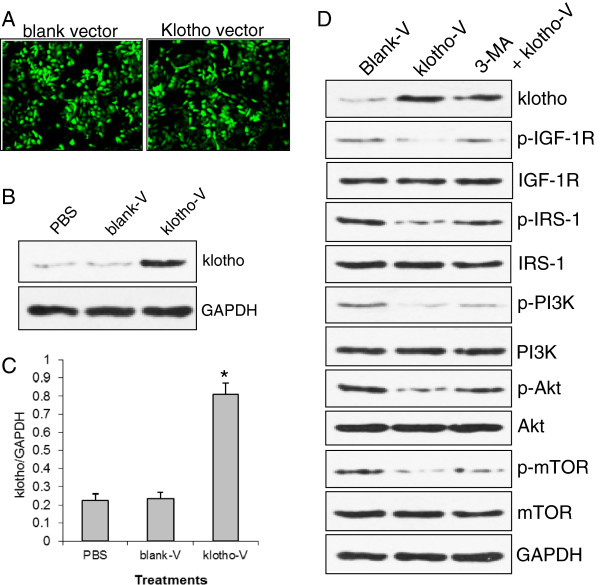
**Klotho inhibited phosphorylation of proteins. A**) Klotho expression in GC-7901 cells transfected with klotho expression vector. **B**) Representative of Western blot of klotho protein expression in GC-7901 cell. **C**) Relative klotho levels in **B**). **D**) Western blot of protein expression and phosphorylation in GC-7901 cell. p-IGF-1R: phospho-IGF-1 receptor; p-Akt: phospho-Akt; p-PI3K: phospho-PI3K; p-IRS-1: phospho-IRS-1; p-mTOR; phospho-mTOR; klotho-V: klotho expression vector; blank-V: blank vector.

To further validate the role of klotho gene, we constructed a vector overexpressing *klotho* gene and delivered it into GC-7901 cells. The transfection of *klotho* gene expression vector can establish 70–80% of efficacy in GC-7901 cells (Figure 5A, indicated by GFP expression). Western blot showed that GC-7901 cells transfected with *klotho* gene expression vector exhibited over 4-fold increase in klotho protein expression compared to cells transfected with blank vector (Figure 5B, C). We further tested the changes in signal molecules in GC-7901 cells transfected with *klotho* expression vector and treated with or without autophagy inhibitor 3-MA (Figure 5D). Restoration of klotho expression significantly decreased the levels of phospho-IGF-1R, phospho-IRS-1, phospho-PI3K, phospho-Akt1, and phospho-mTOR proteins, but had no effect on the total protein levels. 3-MA significantly blocked the effect of klotho gene expression (Figure 5D).

### Klotho plays a role in both apoptosis and autophagy

**Figure 6:**
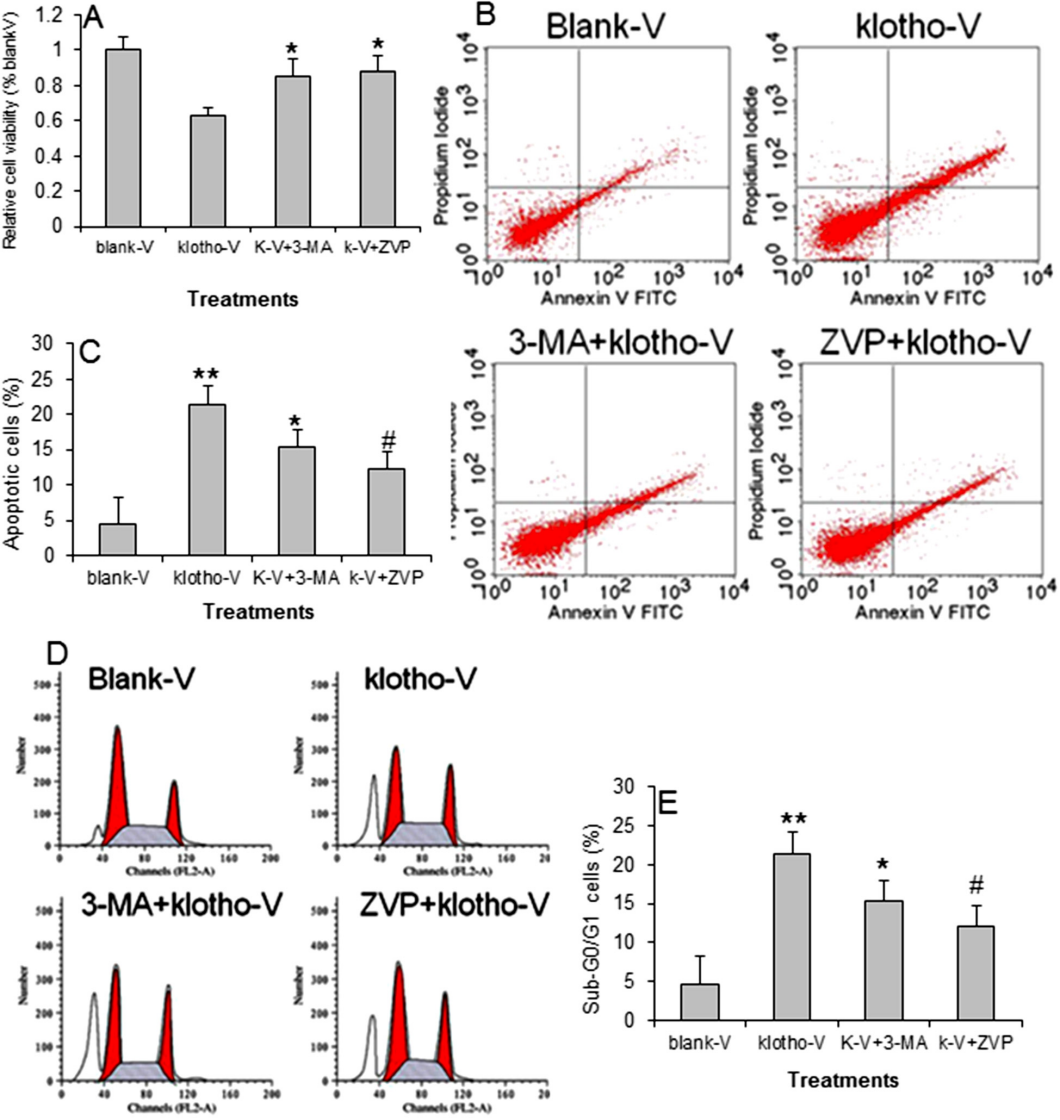
**The roles of apoptosis and autophagy inhibitors on apoptosis and cell cycle. A**) Relative cell viability in GC-7901 cell transfected with blank vector (blank-V), klotho expression vector (klotho-V), or klotho vector plus 3-MA (k-V + 3-MA), or Z-VAD-PMK (k-V + ZVP) incubation. ^*^p < 0.01 *vs. *klotho-V. N = 5. **B**) Flow cytometry of cell apoptosis in GC-7901 cells transfected with blank vector (blank-V) or klotho expression vector (klotho-V), transfected with klotho expression vector plus 3-MA (K-V + 3-MA), or Z-VAD-PMK (k-V + ZVP) incubation. **C**) Percentage of apoptotic cells in **B**). ^**^p < 0.001 *vs. *blank-V, ^*^p < 0.05 *vs. *klotho-V. ^#^*p *< 0.01 *vs. *klotho-V. N = 5. **D**) Flow cytometry of cell cycle analysis. **E**) Percentage of sub-G0/G1 cells in **D**). ^**^p < 0.001 *vs. *blank-V, ^*^p < 0.05 *vs. *klotho-V. ^#^*p *< 0.01 *vs. *klotho-V. N = 5.

**Figure 7:**
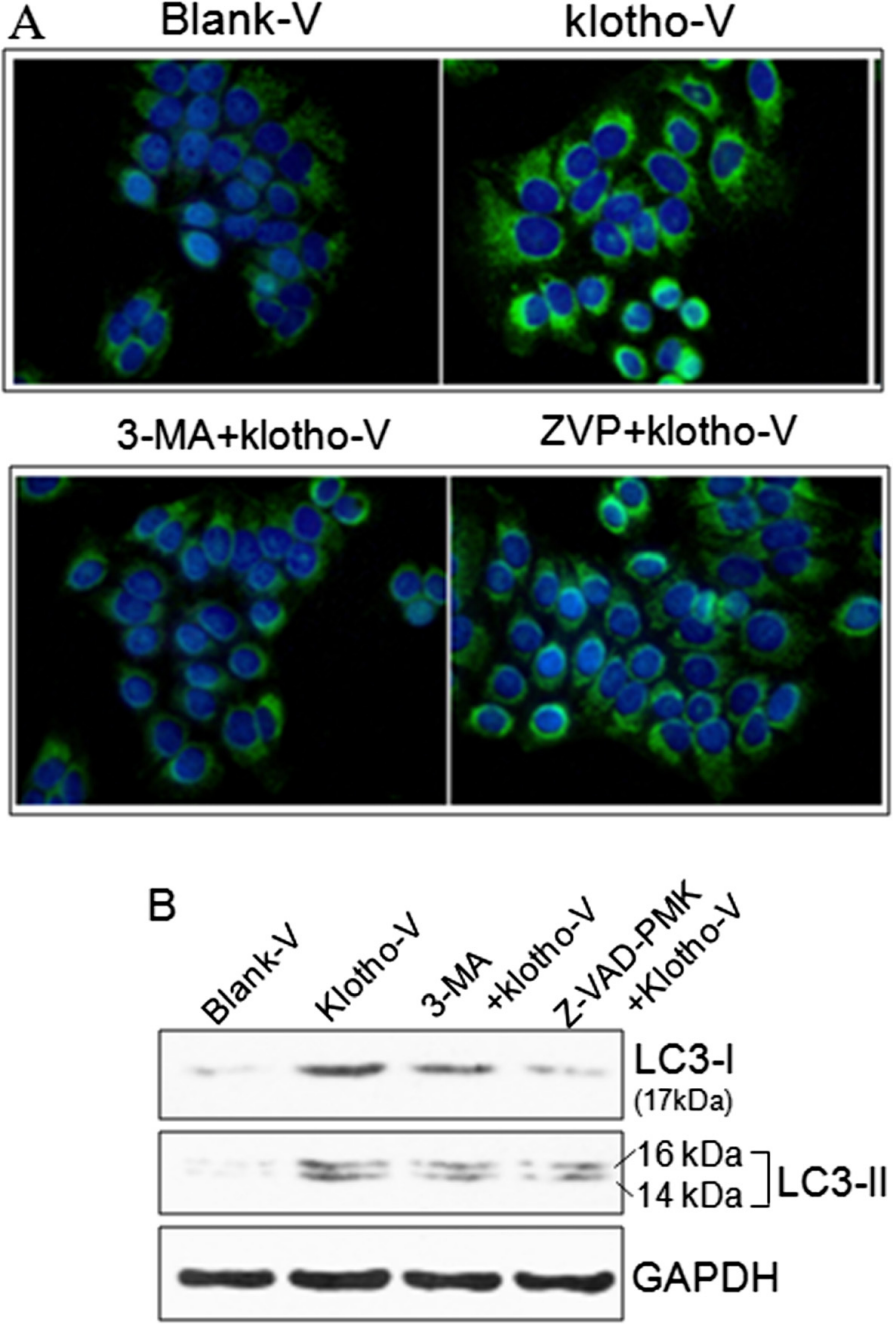
**The roles of apoptosis and autophagy inhibitors on autophagy. A**) Immunofluorence staining of LC3-II expression. The LC3-II positive staining (green) located in the cytoplasm. The blue nuclear was stained by DAPI. GC-7901 cells were transfected with blank vector (blank-V), klotho expression vector (klotho-V), or klotho vector plus 3-MA (k-V + 3-MA), or Z-VAD-PMK (k-V + ZVP). **B**) Western blot of LC3-I and LC3-II expression.

To investigate the involvement of klotho on GC cell apoptosis and autophagy, the *klotho* expression vector-transfected GC-7901 cell was treated with apoptosis inhibitor Z-VAD-PMK and autophagy inhibitor 3-MA. The CCK-8 assay showed that restoration of *klotho* gene expression significantly decreased cell viability in GC-7901 cell. Both the apoptosis and autophagy inhibitors blocked the role of klotho on cell viability (Figure 5A). Also, both the autophagy and apoptosis inhibitors significantly antagonized the role of klotho in inducing apoptosis (Figure 6B, C) and arresting cell cycle (Figure 6D, E). Both the autophagy and apoptosis inhibitors partially decreased klotho-induced LC3-II expression detected by immu-nofluorence (Figure 7A) and LC3-I and LC3-II expression detected by Western blot (Figure 7B).

## Discussion

The *Klotho* gene has been demonstrated to be a tumor suppressor in several tumors, including gastric cancer. However, the signaling pathways by which klotho may inhibit GC cell survival and tumor growth have not been reported. In this study, we validated that the absence of *klotho* gene expression in GC cells is caused by hypermethylation of klotho promoter. Restoration of klotho expression inhibited cell proliferation and induced apoptosis and autophagy in GC through downregulating IGF-1R, IRS-1, PI3K, Akt, and mTOR phosphorylation.

Consistent with previous reports in GC [11] and other cancers [12,13], restoration of *klotho* gene expression induced cell cycle arrest and apoptosis in GC cells. However, the signaling pathways involved in the tumor suppressive roles of klotho in gastric cancer cells have not been clearly elucidated. Klotho is observed in several tumors to induce cell apoptosis and inhibit tumor growth through inhibiting insulin/IGF-1 signaling [14-16] by inhibiting the tyrosine phosphorylation of IGF-1 receptor [17]. IRS-1 is an adaptor of IGF-1R, which plays a key role in transmitting signals from the insulin/IGF-1 receptors to intracellular pathways, including PI3K/Akt/mTOR pathway. Tyrosine phosphorylation of IGF-1R induces the cytoplasmic binding of IRS-1 to IGF-1R and phosphorylation of multiple tyrosine residues in IRS-1. This enables IRS-1 to activate PI3K pathway[18]. In this study, we revealed that restoration of *klotho* gene expression significantly reduced IGF-1R, IRS-1, PI3K, Akt, and mTOR phosphorylation in GC cells. The PI3K-Akt-mTOR pathway plays a pivotal role in the regulation of apoptosis in many cell types. We therefore propose that klotho inhibits IGF-1 receptor phosphorylation, which subsequently inhibits IRS-1 phosphorylation and PI3K-Akt-mTOR signaling. It has been revealed that inhibition of PI3K-Akt-mTOR signaling induced cell cycle G0 and G1 arrest in tumor cells [19].

Autophagy is a physiological mechanism to scavenge toxins and damaged cellular components produced in cells in response to different stresses. However, overactive autophagy will cause autophagic cell death [20,21]. Autophagy is considered to be a major way to kill apoptosis-resistant tumor cells [8]. However, how the autophagy signaling is activated remains to be fully elucidated. There is increasing evidence that tumor suppressor genes promote autophagy [11]. In this study, we detected a low rate of LC3-II/LC3-I expression in the GC cells and restoration of klotho expression significantly increased the ratio. In addition, the autophagy inhibitor 3-MA blocked klotho-induced increases in LC3-II/LC3-I expression. This suggested that klotho induces autophagy in GC. Thus, klotho functions as a tumor suppressor by inhibiting apoptosis and autophagy in GC. However, how klotho exerts roles on both the apoptosis and autophagy in tumor cells is unclear.

The growth factor signaling that involves in the insulin/IGF-1-PI3K-Akt-mTOR pathway has been revealed to regulate cell autophagy through the insulin receptor [22]. In addition, it has been revealed that activation of insulin/IGF signaling can suppress the autophagic-lysosomal pathway [23,24]. Furthermore, the klotho protein functions as a circulating hormone that represses intracellular signals of insulin and IGF-I [17,25]. This suggests that the klotho-IGF-I-PI3K-Akt-mTOR signaling pathway may also be involved in the regulation of autophagy in GC. Indeed, in this study, restoration of *klotho* gene expression induced apoptosis and autophagy as well as inhibiting IGF-1R, IRS-1, PI3K, Akt, and mTOR phosphorylation. Moreover, autophagy inhibitors significantly blocked klotho-induced apoptosis, while apoptosis inhibitor blocked klotho-induced autophagy in GC cells. At the same time, these inhibitors blocked IGF-1R, IRS-1, PI3K, Akt, and mTOR phosphorylation. This suggests that klotho-IGF-1R/IRS-1-PI3K-Akt-mTOR pathway may be involved in both apoptosis and autophagy. Therefore, inhibition of apoptosis though this pathway will also impair autophagy. However, the apoptosis inhibitor cannot completely block klotho-induced authophagy and the same applies to the autophagy inhibitor. This implicates that klotho-induced apoptosis and autophagy have different death pathways.

## Conclusion

In this study, klotho was identified a tumor suppressor, which inhibited tumor cell proliferation, induced cell apoptosis and autophagy in GC. The tumor suppressive role of klotho may be initiated by downregulation of IGF-1 receptor phosphorylation, and subsequent decreases in IRS-1, PI3K, Akt, and mTOR phosphorylation. Our study highlighted the central role of klotho in GC cell survival and suggested that *klotho* gene is an ideal target for developing agent for GC therapy.

## Materials and methods

### Cell culture

MNK-45, AGS, and GC-7901 cells are human gastric cancer cell lines, and GES-1 is a normal gastric epithelial cell line. All cells were obtained from the Shanghai Cell Bank, Chinese Academy of Sciences (Shanghai, China). Cells were cultured in either RPMI 1640 with 10% fetal bovine serum, 100 units/mL penicillin, and 100 μg/mL streptomycin at 37°C, 5% CO_2_.

### RT-PCR of Klotho gene expression

Cultured cells were homogenized in Trizol reagent (Invitrogen, Carlsbad, CA). Total RNA was isolated following the user manual. Reverse transcription was performed using First Strand cDNA Synthesis Kit (Fermentas China, Shenzhen, China). *Klotho* gene fragment was amplified using forward primer: 5^′^- CACGGCAAGGGTGCGTCCAT -3^′^ and reverse primer: 5^′^-TCGCGCCCACGAGATGGAGA-^′^3. The *GAPDH* gene was amplified using forward primer: 5^′^-CTCATGACCACAGTCCATGC-3^′^ and reverse primer: 5^′^-TTCAGCTCTGGGATGACCTT-3^′^. PCR products were visualized on 1.5% agarose gel containing 0.5 μg/ml of ethidium bromide.

### Genomic DNA isolation, sodium bisulfite treatment and PCR amplification

QIAamp DNA Mini Kit (QIAGEN, Valencia, CA, USA) was used to extract genomic DNA from cultured cells by following the user manual. For bisulfite treatment, EZ DNA Methylation-Gold Kit (ZYMO RESEARCH, Orange, CA, USA) was used. After purification, methylated genomic DNA was subjected to PCR amplification of *Klotho* gene promoter. The methylated DNA was amplified by Klotho(M)-F: 5^′^-ATGAATTTGAGCGTTTACGAAAC-3^′^, and Klotho(M)-R 5^′^-ACTCCGCTAACAATAATTACCTACG-3^′^ primers, while the unmethylated DNA was amplified by Klotho(U)-F: 5^′^-ATGAATTTGAGTGTTTATGAAATGT-3^′^, and Klotho(U)-R: 5^′^-TCCACTAACAATAATTACCTACAAA-3^′^ primers. The amplified fragments were 219 bp.

### Western blot

The anti-klotho, anti-Akt, anti-phospho-Akt1, anti-IGF-IR, anti-phospho-IGF-IR, anti-GAPDH, and HRP-conjugated second antibodies were purchased from Santa Cruz Biotechnology (Santa Cruz, CA, USA). The anti-LC3C-I antibody (Cat#: 6976–1) was purchased from Epitomics (Burlingame, CA, USA). The anti-LC3B-II (Cat#: 3868), anti-IRS, anti-phospho-IRS, anti-PI3K, anti-phospho-PI3K, and anti-phospho-mTOR antibodies were purchased from Cell Signaling Technology (Danvers, MA, USA). Protein concentrations were measured using BCA Protein Assay kit (Beyotime, Shanghai, China). Western blot was performed as previously described [26]. Briefly, 20 to 30 μg of total protein were loaded onto a 10 or 12% SDS-PAGE gel and transferred to nitrocellulose membranes. After blocking with 5% non-fat milk for 1 hour, membranes were incubated with primary antibody for 2 hrs at room temperature or overnight at 4°C and subsequently incubated with HRP-labeled secondary antibody (1:2,000 dilution) for 2 hrs at room temperature. Reactive proteins were detected using chemiluminescent reagents (Pierce, Rockford, IL, USA). To control for loading efficiency, the blots were stripped and reprobed with GAPDH antibody. Expressions of all proteins were evaluated relative to GAPDH expression.

### Cell viability assay

Cell Counting Kit-8 (CCK-8) (Beyotime, Shanghai, China) allows sensitive colorimetric assays for cell viability. Briefly, GC-7901 cells were seeded into 96-well plates at 1 × 10^4^ cells per well 24 hrs before transfection. Cells were transfected with klotho expression vector, blank vector, or no vector (PBS) using lipofectamine 2000 according to the user manual (Invitrogen, Grand Island, NY, USA). Cells were then continually cultured in growth medium for 72 hrs. Ten μl of reagent provided with the kit were added to the cells and incubated for 1 h. Cell viability was assessed using the microplate reader at 450 nm. All results were normalized to OD values measured from an identically conditioned well with only growth medium.

### Flow cytometry assay

GC-7901 cells were seeded in 10-cm dishes at a density of 2×10^6^ cells per dish. After cells reached 70% confluency, cells were transfected with klotho expression vector, blank vector, or PBS as described above. Cells were then trypsinized and suspended with 500 μl of binding buffer containing 5 μl of Annexin V-FITC and 5 μl of Propidium Iodide (Abcam, Cambridge, MA, USA). After incubation in the dark for 1 hour, cells were subjected to flow cytometry assay.

### Construction of klotho gene expression vector

The *klotho* gene was amplified from a cDNA library established from GES-1 cells. The open read frame (ORF) of klotho cDNA sequence was amplified by a forward primer containing Bgl II sequence (italic): ACTCAGATCTGAGCCGGGCGACGGCGCGCAGA and reverse primer containing a BamHI site (italic): CGGTGGATCCCCTATTTGTAACTTCTTCTGCC. The amplified *klotho* ORF was then cloned into pZsGreen1-C1 vector at Bgl II/Bam HI sites (Clontech, Mountain View, USA). The *klotho* ORF was fused with GFP at the C-terminal of GFP. The pZsGreen1-C1 vector without insertion was used as a blank vector control.

### Immunofluorescence

To identify the location and expression of LC3-II protein, we performed immunofluorescent staining in GC-7901 cells. Briefly, cells in 24-well plates were fixed by 10% paraformaldehyde for 30 min at 4°C. After cells were rinsed with PBS for 3 × 5 min, they were permeabilized with 0.5% Triton X-100 for 15 min. After a light rinse with PBS for 3 times, cells were incubated with 10 mM citrate buffer (pH 3.0) for antigen retrieval for 30 min and then incubated with 10% goat serum for 1 h to block nonspecific staining. Subsequently, the cells were incubated with rabbit-anti-LC3-II antibody (Cell Signaling Technology, Danvers, MA, USA) overnight at 4°C. After washing cells with PBST (PBS plus 0.05%Tween-20) for 3 × 5 min, cells were continually incubated with goat-anti-rabbit, FITC conjugated antibody (1:600, Cell Signaling Technology) for 1 h at room temperature, and then cells were washed with PBST and mounted with anti-fade medium. The staining was examined using a fluorescence microscope.

### Cell treatments with autophagy and apoptosis inhibitors

GC-7901 cells at 70% confluency were transfected with klotho expression vector, blank vector, or PBS as described above. Cells transfected with klotho expression vector or PBS were incubated with 10 mM of autophagy inhibitor 3-methyladenine (3-MA) or 20 μM of apoptosis inhibitor Z-VAD-FMK for 24 hours. Cells were then harvested for Western blot and/or flow cytometry assay.

### Statistical analysis

Data was analyzed using the SPSS 13.0 (statistical package for the Social Sciences Version 13.0). Two samples were compared using student t-test. A *p* < 0.05 was considered statistically significant.

## Abbreviations

GC: Gastric cancer; PTEN: Phosphatase and tensin homolog; IGF-1: Insulin/ insulin-like growth factor-1; IRS-1: Insulin receptor substrate 1; PI3K: Phosphoinositide 3-kinase; LC3: Microtubule-associated protein light chain 3; Akt: Protein Kinase B; mTOR: Mammalian target of rapamycin; 5-Aza: 2^′^-deoxy-5-azacytidine; 3-MA: 3-methyladenine.

## Competing interests

All authors declared no conflict of interest.

## Authors’ contributions

BX: Experiment design, acquisition of data, analysis and interpretation of data, preparation of manuscript. JZ: acquisition of data. GS: acquisition of data. DL: conception and design, revising manuscript critically for important intellectual content. JZ: analysis and interpretation of data. JC: conception and design. LY: conception and design, final approval of manuscript. All authors read and approved the final manuscript.
